# Regulatory B Cells Inhibit Cytotoxic T Lymphocyte (CTL) Activity and Elimination of Infected CD4 T Cells after *In Vitro* Reactivation of HIV Latent Reservoirs

**DOI:** 10.1371/journal.pone.0092934

**Published:** 2014-04-16

**Authors:** Basile Siewe, Jennillee Wallace, Sonya Rygielski, Jack T. Stapleton, Jeffrey Martin, Steven G. Deeks, Alan Landay

**Affiliations:** 1 Rush University Medical Center, Department of Immunology and Microbiology, Chicago, Illinois, United States of America; 2 Iowa City Veterans Affairs Medical Center and the University of Iowa, Departments of Internal Medicine, Microbiology and Immunology, Iowa City, Iowa, United States of America; 3 HIV/AIDS Division, San Francisco General Hospital, University of California San Francisco (UCSF), San Francisco, California, United States of America; 4 FC Donders Chair, Division of Pharmacology, Utrecht Institute of Pharmaceutical Sciences, Faculty of Science, Utrecht University, Utrecht, The Netherlands; New York University, United States of America

## Abstract

During HIV infection, IL-10/IL-10 receptor and programmed death-1 (PD-1)/programmed death-1-ligand (PD-L1) interactions have been implicated in the impairment of cytotoxic T lymphocyte (CTL) activity. Despite antiretroviral therapy (ART), attenuated anti-HIV CTL functions present a major hurdle towards curative measures requiring viral eradication. Therefore, deeper understanding of the mechanisms underlying impaired CTL is crucial before HIV viral eradication is viable. The generation of robust CTL activity necessitates interactions between antigen-presenting cells (APC), CD4^+^ and CD8^+^ T cells. We have shown that in vitro, IL-10^hi^PD-L1^hi^ regulatory B cells (Bregs) directly attenuate HIV-specific CD8^+^-mediated CTL activity. Bregs also modulate APC and CD4^+^ T cell function; herein we characterize the Breg compartment in uninfected (HIV_NEG_), HIV-infected “elite controllers” (HIV_EC_), ART-treated (HIV_ART_), and viremic (HIV_vir_), subjects, and in vitro, assess the impact of Bregs on anti-HIV CTL generation and activity after reactivation of HIV latent reservoirs using suberoylanilide hydroxamic acid (SAHA). We find that Bregs from HIV_EC_ and HIV_ART_ subjects exhibit comparable IL-10 expression levels significantly higher than HIV_NEG_ subjects, but significantly lower than HIV_VIR_ subjects. Bregs from HIV_EC_ and HIV_ART_ subjects exhibit comparable PD-L1 expression, significantly higher than in HIV_VIR_ and HIV_NEG_ subjects. SAHA-treated Breg-depleted PBMC from HIV_EC_ and HIV_ART_ subjects, displayed enhanced CD4^+^ T-cell proliferation, significant upregulation of antigen-presentation molecules, increased frequency of CD107a^+^ and HIV-specific CD8^+^ T cells, associated with efficient elimination of infected CD4^+^ T cells, and reduction in integrated viral DNA. Finally, IL-10-R and PD-1 antibody blockade partially reversed Breg-mediated inhibition of CD4^+^ T-cell proliferation. Our data suggest that, possibly, via an IL-10 and PD-L1 synergistic mechanism; Bregs likely inhibit APC function and CD4^+^ T-cell proliferation, leading to anti-HIV CTL attenuation, hindering viral eradication.

## Introduction

CD8 cytotoxic T lymphocyte (CTL) activity is critical in controlling viral replication during HIV infection (reviewed in [1]). Individuals who naturally control HIV replication in the absence of therapy (“elite controllers”, HIV_EC_) often exhibit robust CTL activity [2], [3]. In contrast, CTL function is severely attenuated in individuals who do not control HIV, and this impaired CTL activity is not restored even with successful ART [2].

In ART-treated HIV-infected subjects (HIV_ART_), viral replication is suppressed but the virus persists because early in infection HIV establishes latent reservoirs and upon ART interruption, HIV replication is detected [4], [5]. The establishment of stable latent reservoirs [6] dictates lifelong ART treatment associated with financial cost and potential toxicity, thus, therapies leading to HIV eradication are urgently warranted. Recent studies have focused on using small molecules that, unlike antibodies, reactivate the latent reservoirs without inducing unrestrained T cell activation [7]. However, data from a seminal study by Shan et al [8] indicate that reactivating the viral reservoir using the FDA-approved histone deacetylase inhibitor (HDACi), suberoylanilide hydroxamic acid (SAHA) was not associated with the death of infected CD4^+^ T cells as was previously hypothesized. In contrast, Shan et al determined that post reactivation of latent reservoirs an efficient CTL response was indispensable to clear infected cells. Since CTL responses are impaired in HIV_ART_ subjects, Margolis and Hazuda [9] suggest that HIV eradication would require a dual approach: reactivation of the latent reservoir without inducing global activation, concomitant with strategies to boost the immune response, specifically anti-HIV CTL responses. This indicates that understanding and delineating the mechanisms underlying CTL impairment in ART-treated HIV-infected subjects is critical before HIV eradication becomes viable. We have shown that in HIV_ART_ subjects, IL-10 expressing regulatory B cells (Bregs, CD19^+^CD24^hi^CD38^hi^) attenuate anti-HIV CTL activities in vitro by directly inhibiting the proliferation of antigen-specific cytotoxic CD8^+^ T cells in a partially IL-10 dependent manner [10]. Similarly, Das et al report that CD19^+^CD24^hi^CD38^hi^ Bregs impair CTL activity during chronic Hepatitis B virus infection [11]. However, interactions between proliferating CD4^+^ T cells and antigen presenting cells (APC) are also critical in generating effective CTL responses [12]. Interestingly, studies show that activated B cells negatively regulate CD4^+^ T cell proliferation and APC function [13]–[15], indirectly attenuating the generation of effective CTL, however this has not been investigated in human viral infections.

In this study, the goal was twofold: a comprehensive characterization of the Breg compartment in HIV-infected subjects including “elite controllers” and assessing the anti-HIV CTL inhibitory role for Bregs in the clinically relevant context of latent reservoir reactivation. We determine phenotypic and functional similarities between Bregs from HIV_EC_ and HIV_ART_ subjects. Further, in vitro after SAHA treatment, Bregs directly and indirectly attenuate anti-HIV CTL activity. The mechanism likely involves modulation of mediators of CTL generation via IL-10 and/or PD-L1. To our knowledge, these data represent the first report demonstrating possible mechanisms by which Bregs directly attenuate HIV-specific CTL generation and function in a human viral infection with potential therapeutic importance in eradication of HIV.

## Results

### HIV-infected “Elite Controllers” (HIV_EC_) and Uninfected (HIV_NEG_) Individuals have Comparable Breg-frequency

**Figure 1:**
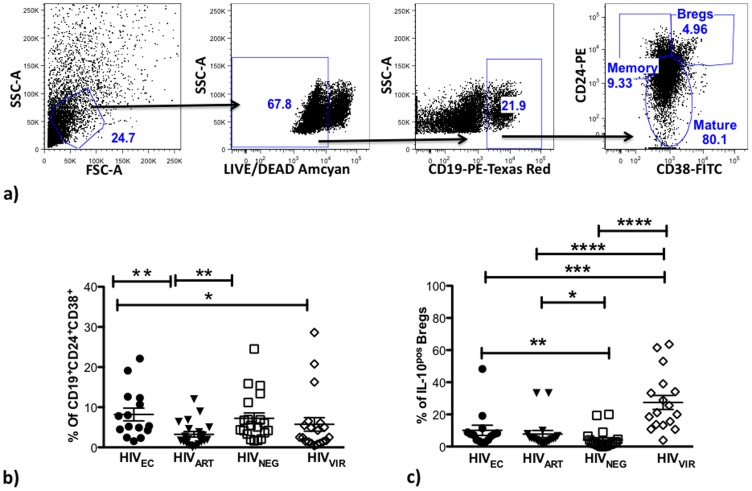
HIV_EC_ and HIV_NEG_ have comparable Bregs frequency. PBMC from HIV_EC_ (n = 15), HIV_ART_ (n = 20), HIV_NEG_, (n = 20) and HIV_VIR_, (n = 17) were cultured for 48H and during the final 5H supplemented with PMA (25 ng/ml), Ionomycin (1 ug/ml), Brefeldin A (1∶100) and by flow cytometry, the (**a,b**) frequency of CD19^+^CD24^hi^CD38^hi^ Bregs and (**c**) IL-10-positive Bregs (intracellular cytokine staining) determined. p values for differences as calculated by Mann Whitney test two-tailed t test (Graphpad Prism software) are indicated; * = p<0.05, ** = p<0.01, lines indicate mean with SEM.

We have previously shown in vitro that after stimulation with HIV peptides, Bregs from HIV_ART_ subjects directly attenuate the proliferation of HIV-specific CD8^+^ T cells and anti-CTL activities in a partially IL-10-dependent manner [10]. We characterized Bregs (gating strategy shown in Figure 1a) from HIV_EC_ (n = 15); healthy controls HIV_NEG_ (n = 20), HIV_ART_ (n = 20), and viremic HIV-infected subjects (HIV_VIR_) (n = 17). Breg frequencies were similar between HIV_EC_ and HIV_NEG_ groups, and were lower in the HIV_VIR_ group, (Figure 1b, p = 0.04 for HIV_VIR_ versus HIV_EC_, p = 0.06 for HIV_VIR_ versus HIV_NEG_). Surprisingly, Breg frequencies were lowest in the HIV_ART_ group (Figure 1b, p = 0.0015 for HIV_ART_ versus HIV_EC_, p = 0.01 for HIV_ART_ versus HIV_NEG_). By intracellular cytokine staining (Figure 1c) we determined that a significantly higher percentage of Bregs in HIV_VIR_ subjects were IL-10 positive compared to Bregs from HIV_EC_ (p = 0.0006), HIV_ART_ (p<0.0001) and HIV_NEG_ (p<0.0001) subjects. Further, Bregs from HIV_EC_ and HIV_ART_ subjects exhibit comparable frequencies of IL-10 positive cells, significantly higher than in HIV_NEG_ subjects (Figure 1c, p = 0.0043 and p = 0.0119 respectively).

### Breg-Depleted SAHA-Treated PBMC from HIV_EC_ and HIV_ART_ Individuals Exhibit Heightened Frequency of HIV-specific Cytotoxic CD8^+^ T cells

**Figure 2:**
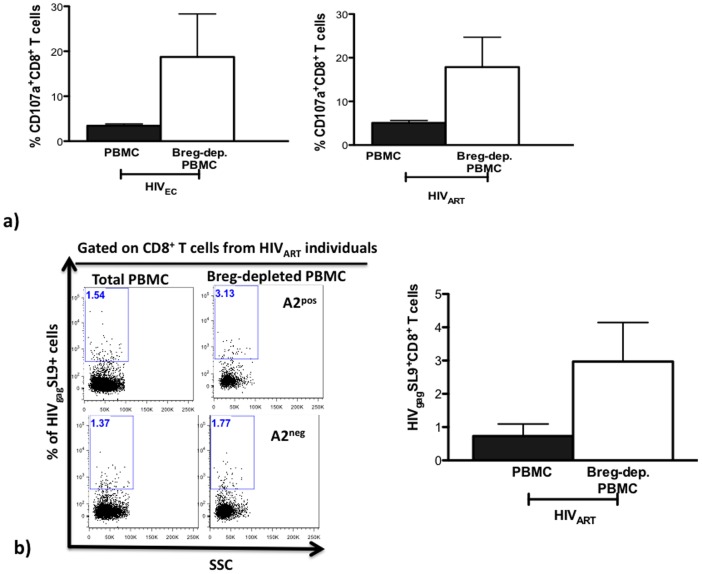
SAHA treated Breg-depleted PBMC from HIV_EC_ and HIV_ART_ exhibit higher frequencies of anti-HIV CTL-competent CD8^+^ T cells. (**a**) 500 nM SAHA-treated total or Breg-depleted PBMC from HIV_EC_ (n = 4) and HIV_ART_ (n = 6) were cultured for 4 days and the frequency CD107a^+^CD8^+^ T cells determined by flow cytometry. (**b**) After 4 days in culture, by flow-cytometry using an HLA-A*0201 MHC-I HIV Dextramer® (Immudex), the frequency of HIV_gag_SL9^+^ CD8^+^ T cells was determined; left panel depicts representative dot-plots demonstrating specific binding and right panel shows the summary of results (A2^pos^ = HLA-A*2 positive, A2^neg^ = HLA-A*2 negative). p values for differences as calculated by paired one-tailed t test (Graphpad Prism software) are indicated.

We next investigated the impact of Bregs on CTL activity in a clinically relevant setting. Results from in vitro and in vivo studies have demonstrated that treatment with the histone deacetylase inhibitor (HDACi), suberoylanilide hydroxamic acid (SAHA) leads to reactivation of HIV latent reservoirs [16]–[18]. In SAHA-treated PBMC from HIV_EC_, Breg depletion led to enhanced CD107a expression (averagely, 451% increased expression) on CD8^+^ T cells of all subjects investigated (n = 4, Figure 2a, left panel). There was an increase in CD107^+^CD8^+^ T cells (averagely, 251% increased expression) in 80% of the HIV_ART_ subjects (5 of 6, Figure 2a, right panel). This observed increase in the frequency of cytotoxic CD8^+^ T cells was also associated with an increased frequency of CD8^+^ T cells expressing HIV_gag_ CTL-associated SL9 epitope [19]–[21] (averagely 282% increased expression) in the HIV_ART_ (n = 4) subjects studied (Figure 2b).

### In Breg-depleted SAHA-Treated PBMC from HIV_EC_ and HIV_ART_ Subjects, Heightened Frequency of CTL-Competent CD8^+^ T cells is Associated with Efficient Elimination of HIV Infected CD4^+^ T Cells and Reduction of Viral DNA

**Figure 3:**
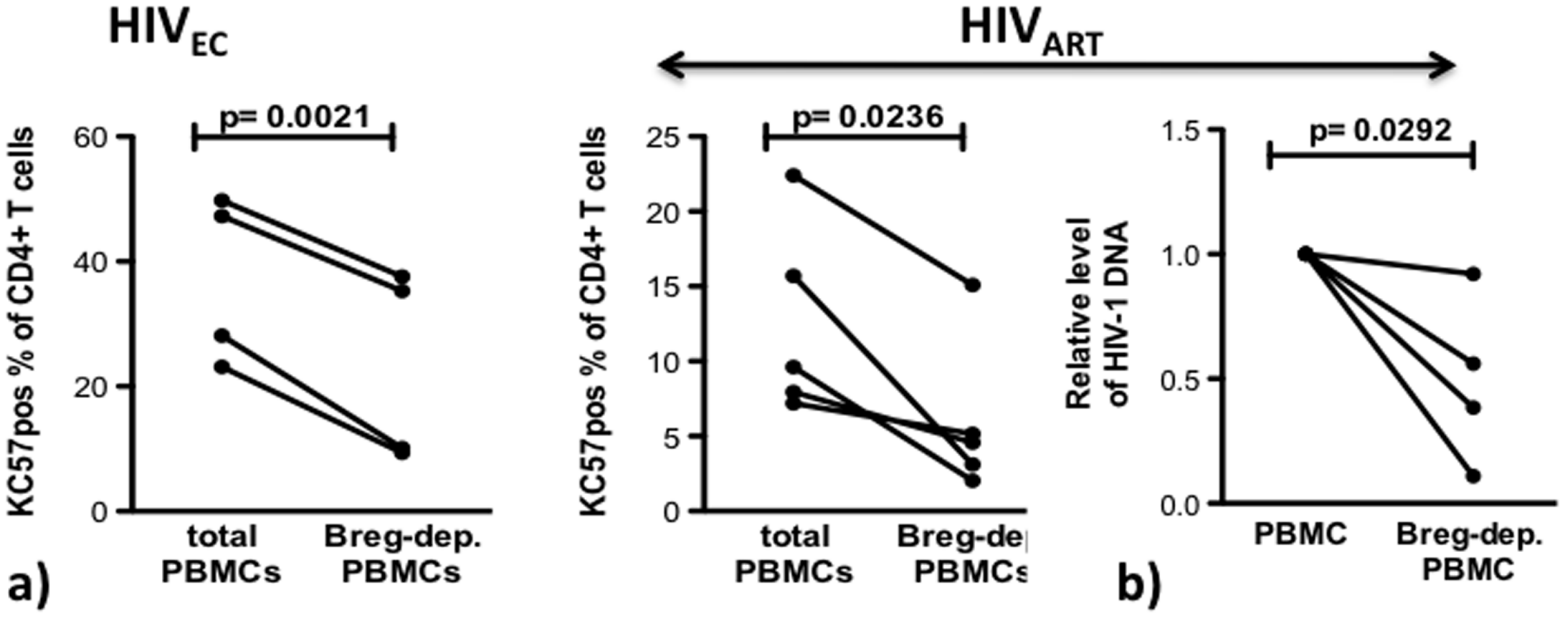
Association between elevated frequency of CTL–competent T cells, clearance of infected CD4^+^ T cells and reduced viral DNA. (**a**) 500 nm SAHA-treated total or Breg-depleted PBMC from HIV_EC_ (n = 4) and HIV_ART_ (n = 5) were cultured for 4 days and the frequency of infected CD4^+^ T cells was determined by binding to KC57-antibody. (**b**) In HIV_ART_ subjects (n = 5), relative levels of HIV DNA between SAHA-treated total or Breg-depleted PBMC were determined by qPCR with LTR hybridizing primers after 4 days in culture. p values as calculated by paired two-tailed t test (Graphpad Prism software) are indicated.

After determining that Breg depletion resulted in elevated frequency of CTL-competent CD8^+^ T cells (identified by CD107a expression), we investigated if this leads to enhanced clearance of infected cells. By intracellular cytokine staining (Figure 3a), we determined that Breg depletion led to a significantly reduced frequency of infected CD4^+^ T cells in PBMC from HIV_EC_ (p = 0.0021) and HIV_ART_, (p = 0.0236) (HIV-1 core protein positive cells, Figure S1). Finally, by quantitative RT-PCR with primers hybridizing in the HIV LTR we determined that Breg depletion was associated with a significant decrease (p = 0.0292) in viral DNA in SAHA-treated PBMC from HIV_ART_ (n = 4) (Figure 3b).

### After SAHA Treatment, Breg Depletion leads to Enhanced Expression of Antigen Presentation Molecules and Heightened Proliferation of CD4^+^ T cells

**Figure 4:**
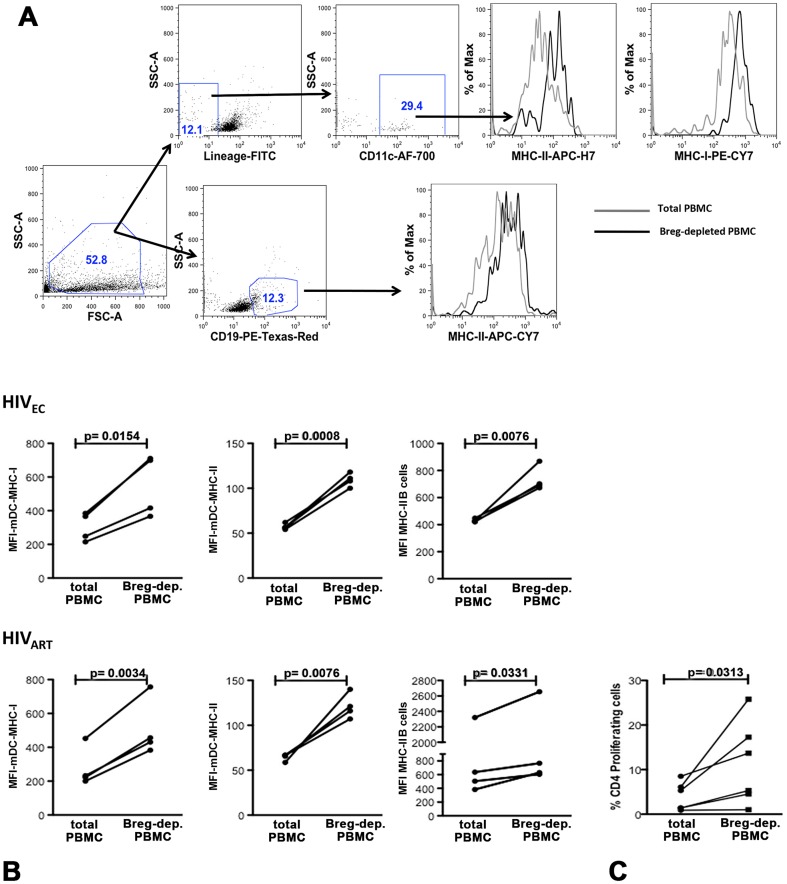
SAHA-treated Breg-depleted PBMC from HIV_EC_ and HIV_ART_ exhibit upregulated expression of antigen-presenting molecules and proliferation of CD4+ T cells. After 4 days in culture, (**a**) the expression of MHC-II and MHC-I/II on B cells and dendritic cells (LIN^−^CD11c^+^HLA-DR^+^) respectively was determined by flow cytometry in (**b**) SAHA-treated total or Breg-depleted PBMC from HIV_EC_, (n = 4, upper panel) and HIV_ART_, (n = 4, lower panel). The gating strategy and representative histogram overlays are depicted in Figure 3a. (**c**) VPD450-proliferation dye labeled total or Breg-depleted PBMC were stimulated with SAHA (500 nM, Figure 1b right panel, n = 5) and after 4 days in culture proliferation of CD4^+^ T cells was determined by flow cytometry. p values for differences in CD4^+^ T cell proliferation as calculated by paired two-tailed t test (Graphpad Prism software) are indicated.

In these SAHA treated samples, we next investigated the underlying mechanisms by which Bregs mediate inhibition of CTL activity. The generation of robust CTL responses requires interactions between antigen presenting cells (APC) and proliferating CD4^+^ T cells [12]. We determined that in the HIV_EC_ subjects, Breg depletion led to enhanced DC expression of MHC-I (p = 0.0154) and MHC-II (p = 0.0008) as well as B-cell expression of MHC-II (p = 0.0076) (Figure 4b). Similarly, in HIV_ART_ subjects Breg depletion led to enhanced DC expression of MHC-I (p = 0.0034), and MHC-II (p = 0.0076) as well as B-cell expression of MHC-II (p = 0.0331) and proliferation of CD4^+^ T cells (Figure 4b,c, p = 0.0313).

### Bregs from HIV_EC_ and HIV_ART_ Individuals Express Elevated PD-L1 Levels and Breg Inhibition of CD4^+^ T-cell Proliferation is Partially PD-L1 and IL-10 Dependent

**Figure 5:**
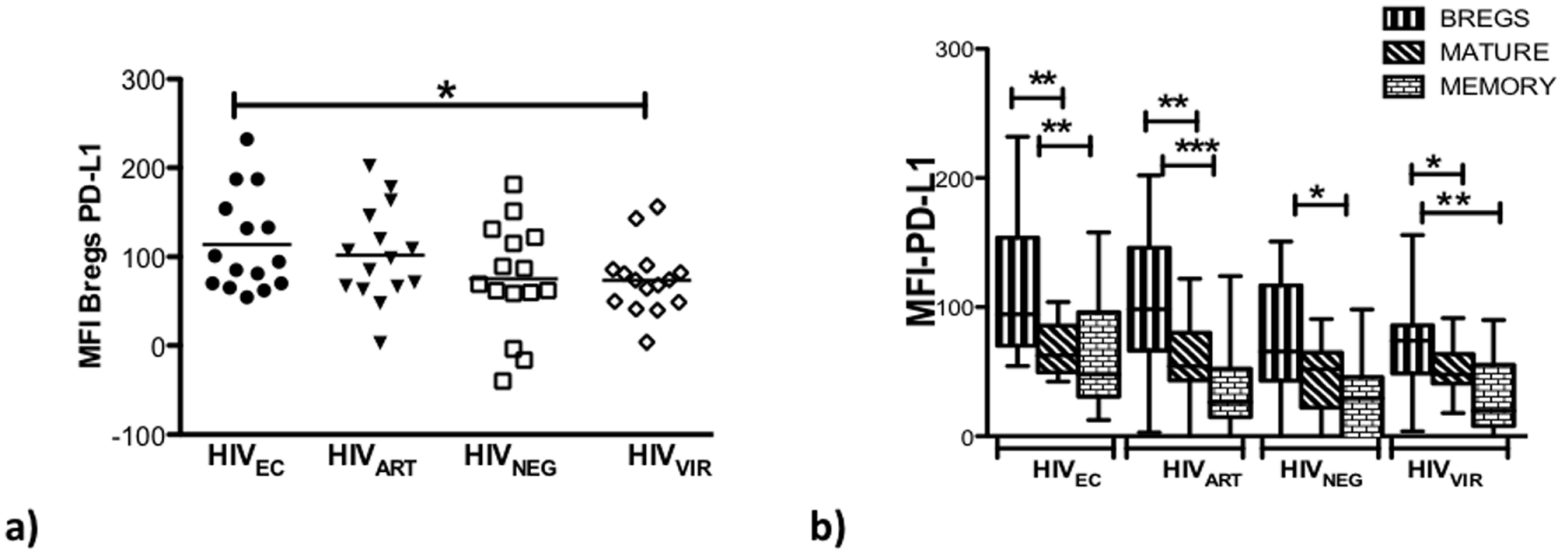
Bregs from HIV_EC_ and HIV_ART_ subjects exhibit comparable endogenous levels of PD-L1. In PBMC (n = 15) of HIV_EC_, HIV_ART_, HIV_NEG_, and HIV_VIR_, endogenous levels of PD-L1 expression on (**a**) Bregs and (**b**) Bregs compared to the other B-cell subsets were determined by flow cytometry. Boxes represent 25^th^ to 75^th^ percentiles, whiskers indicate minimum and maximum values and the lines indicate the median. P values (Graphpad Prism software) are indicated; * = p<0.05, ** = p<0.005, *** = p<0.0005.

We previously demonstrated that TLR activated Bregs upregulate PD-L1 expression [10]. Further, during HIV infection, exhausted PD-1^hi^ T cells significantly contribute to viral persistence [22]–[25] and in vivo PD-1 blockade has been shown to lead to reduction in viral load [26], [27]. We therefore investigated PD-L1 expression levels on Bregs from HIV_NEG_, HIV_VIR_, HIV_AVIR_, and HIV_EC_ (Figure 5a). We determined that Bregs from HIV_ART_, and HIV_EC_ have comparable levels of PD-L1 expression. In contrast, Bregs from HIV_EC_ had higher levels of PD-L1 expression compared to Bregs from HIV_NEG_ (p = 0.0929) and HIV_VIR_ (p = 0.0421). Bregs from HIV_ART_ expressed 34% and 38% more PD-L1 than Bregs from HIV_NEG_ and HIV_VIR_ respectively. Interestingly, in HIV_EC_, HIV_ART_ and HIV_VIR_, compared to other B cell subsets, Bregs expressed significantly higher levels of PD-L1 (Figure 5b).

**Figure 6:**
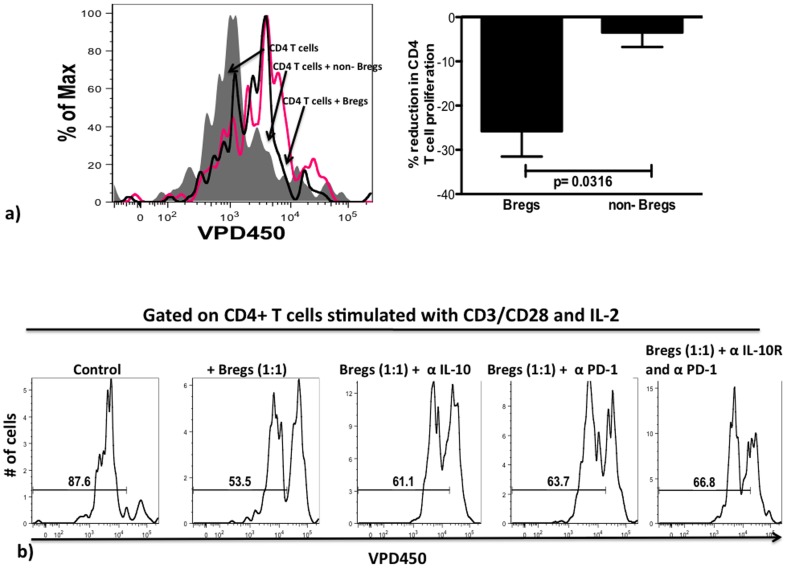
In HIV_ART_ subjects Breg-mediated inhibition of CD4^+^ T cell proliferation is dependent on a synergy between PD-L1 and IL-10. Bregs or non-Bregs B cells were co-cultured for 72 hours with VPD450-labeled FACS-purified CD4^+^ T cells, activated using Anti-Biotin MACSiBead (Miltenyi, T Cell Activation/Expansion Kit) and IL-2 (2 U/ml) and proliferation of CD4+ T cells determined by flow cytometry. (**a**) Right panel, shows a representative overlay and right panel depicts compilation of results, whereby the reduction in proliferation is normalized to CD4^+^ T cell proliferation alone (n = 4). (**b**) In antibody blocking experiments, labeled and activated CD4+ T cells were co-cultured with Bregs and under conditions shown above the representative histograms (n = 4) (α-IL-10 = IL-10R blocking antibody (20 ug/ml, Biolegend), α-PD-1 = PD-1 blocking antibody (10 ug/ml, Biolegend).

To assess the contribution of IL-10 and PD-L1 to Breg-immunoregulation, we employed CD4^+^ T cell proliferation co-culture assays. Purified VPD450-labeled CD4^+^ T cells were stimulated with MACSiBeads (Miltenyi, T cell Activation/Expansion kit) and IL-2, either cultured with medium alone, co-cultured with non-Breg B cells, with Bregs, or co-cultured with Bregs supplemented either with anti-IL-10 receptor (IL-10R) blocking antibody, a PD-1 blocking antibody or both anti-IL-10R and anti-PD-1 blocking antibodies combined, as described elsewhere [22]. We determined that CD4^+^ T cells co-cultured with Bregs proliferated significantly less that those co-cultured with non-Bregs (p = 0.0316, Figure 6a). Further, in the Bregs co-cultures, addition of IL-10R, PD-1 antibody and both IL-10R and PD-1 blocking antibodies led to 14%, 19% and 24% respective increased CD4^+^ T-cell proliferation compared to the control (Figure 6b); comparable results have been reported in similar antibody blocking assays [28].

## Discussion

In vitro data from a seminal study by Shan et al, [8] indicate that despite efficient reactivation of the reservoirs using SAHA, effective clearance of infected cells requires robust anti-HIV CD8^+^ CTL responses, which remain attenuated in ART-treated HIV-infected subjects. Thus understanding the mechanisms underlying the attenuated CTL activity in ART-treated HIV-infected subjects is crucial before viral eradication is feasible. We have shown that in vitro, Bregs inhibit the generation of anti-HIV CTL activity after antigenic stimulation [29], here we provide compelling data indicating that in vitro, after SAHA-mediated reactivation of HIV reservoirs, Bregs exert a similar anti-HIV CTL inhibitory role.

Regulatory B cells (Bregs) have been attributed divergent phenotypic (reviewed in [30]) markers, thus IL-10 production remains the de facto Breg marker. However, our group as well as others have shown that during human viral infections, CD19^+^CD24^hi^CD38^hi^ B cells are highly IL-10 competent and exert a regulatory function [11], [29]. Here we furnish further evidence supporting this notion. We find that amongst B cell subsets, Bregs express the highest levels of immunosuppressive mediators IL-10 and PD-L1. Further, unlike Bregs, a non-Breg subset (CD19^+^CD24^lo^CD38^lo^) did not robustly inhibit CD4^+^ T cell proliferation.

To perform an exhaustive assessment of the Breg compartment during HIV pathogenesis, we used PBMC from HIV-infected “elite controllers”, ART-treated and viremic subjects as well as uninfected subjects as controls. Robust and polyfunctional CTL activities in HIV-infected “elite controllers” have been associated with their ability to control HIV replication without ART [3], [31]. We find that HIV_EC_ and HIV_NEG_ subjects exhibit comparable Breg-frequency that was slightly reduced in HIV_VIR_ subjects and lowest in the HIV_ART_ subjects. Some reports indicate that IL-10-competent B cells are susceptible to apoptosis [32], and we observed that compared to other B cell subsets, Bregs from HIV-infected subjects express significantly higher levels of Annexin V and lower anti-apoptotic Bcl-2 expression (Figure S2), suggesting that Bregs may be more prone to undergo cell death in HIV infected individuals. These data collectively suggest that HIV causes a loss of these cells, and that this loss is not readily reversed with ART.

Regarding the expression of immunosuppressive mediators IL-10 and PD-L1 by Bregs, we determined that Bregs from HIV_EC_ and HIV_ART_ subjects constitutively expressed comparable levels of IL-10 and PD-L1, higher than in HIV_NEG_. However, Bregs from HIV_VIR_ expressed the most IL-10 though lower PD^_^L1 levels that were comparable to Bregs from HIV_NEG_.

We have previously shown in vitro that, Breg inhibition of CD8^+^ T cell proliferation is partially IL-10 dependent [10]. However, our finding that Bregs also express high levels of PD-L1 prompted us to investigate if Bregs also exert their immunoregulatory function via PD-L1-PD-1 interactions. Data from our antibody blockade study, suggest that Bregs-immunoregulatory function likely involves a synergistic effect of IL-10 and PD-L1, comparable to results from a study by Brooks et al [33]. In autoimmune settings, murine Bregs exert immunoregulatory functions via cognate T-cell ligands including CD40, MHCII and IL-21 [34]. However, we did not find any difference in the expression of CD40, MHCII and IL-21R on the Bregs in our subjects (data not shown), indicating key differences between Breg immunoregulatory pathways in autoimmune diseases and viral infections. There is growing interest in blocking the PD-1 pathway as part of an HIV cure strategy [26], [35]. Our data suggest that in the context of long-term effective ART, blocking either PD-1L or PD-1 may result in the generation of effective generation of anti-HIV CTL, and ultimately enhanced clearance of infected cells.

Finally, we assessed the impact of Bregs on anti-HIV CTL activity after SAHA-treatment. We used degranulation (CD107a expression) as the measure of CTL-competency and determined that following reactivation of latent reservoirs using SAHA, Breg-depletion modulated critical mediators of robust CTL generation including antigen presentation and CD4^+^ T cell proliferation [12], [36]. Murine Bregs have been shown to modulate APC function and inhibit CD4^+^ T cell proliferation [13], [14], [37] but to our knowledge this is the first time this has been demonstrated for IL-10-competent Bregs during a human viral infection. In these Breg-depleted samples, the robustness of the CTL response was reflected by the enhanced expression of CTL-competent CD107a^+^ and HIV-specific CD8^+^ T cells. Finally, we determined significantly enhanced clearance of infected CD4^+^ T cells, a significant reservoir of latent HIV [4], [5], [38].

Taken together our results suggest that during HIV infection CD19^+^CD24^hi^CD38^hi^ Bregs represent the predominant IL-10 producing B-cell subset, consistent with previous data suggesting a similar role for Bregs during hepatitis B viral infection [39]. Further, we provide novel evidence indicating that Bregs represent the B-cell subset with highest levels of PD-L1 expression in HIV infection, although more studies are warranted to delineate the patterns of Breg IL-10 and PD-L1 expression. Finally, our results suggest a role for Bregs in attenuating CTL responses after reactivation of HIV latent reservoirs. Ineffective CTL responses present a critical hurdle in the quest for HIV eradication. Thus further elucidation of Breg phenotype and regulation could potentially lead to therapies boosting the anti-HIV responses and HIV eradication during curative measures.

## Materials and Methods

### Ethics Statement and Study Participants

All studies were performed after signed informed written research consent by each study subject. The study was reviewed and approved by the Institutional Review Board of the Rush University Medical Center, University of California—San Francisco (UCSF), and the University of Iowa City VAMC and University of Iowa. HIV-uninfected (HIV_NEG_) subjects had a median CD4 count of 777 cells/µl (range: 380–1487). HIV-infected viremic (HIV_VIR_) subjects had a median CD4 count of 495 cells/µl (range: 240–1136) and median viral load of 40909 copies/ml (range: 11023–3140000). Antiretroviral-treated HIV-infected (HIV_ART_) subjects had a median CD4 count of 435 cells/µl (range: 212–1076) and median viral load of 40 copies/ml (range: 0–800). HIV-infected “Elite” controllers (HIV_EC_) had a median CD4 count of 763 cells/µl (range: 454–1595) and median viral load of 48 copies/ml (range: 20–1226). HIV_EC_ were characterized as HIV-infected subjects capable of maintaining their viral load at <2000 copies/ml without ART as previously described [3].

### Analysis of IL-10 Production by Bregs and Immunophenotyping of PBMCs

To determine endogenous intracellular IL-10 production, PBMC were cultured for 48 hours; during the final 5 hours of incubation the cultures were supplemented with Brefeldin A (1∶100, BD), PMA (50 ng/ml, Invivogen) and Ionomycin (1 ug/ml, Invivogen). After incubation the cells were washed, stained for viable cells (LIVE/DEAD Aqua Fixable Dead Cell Stain Kit, Invitrogen), surface stained, fixed/permeabilized (Fix/Perm Kit BD Biosciences) and stained for intracellular IL-10 (IL-10-AF-647, eBioscience). To determine spontaneous expression of IL-10 by Bregs from HIV-infected individuals and healthy controls, PBMCS were incubated overnight, stimulated for the final 5 hours and stained as described for healthy controls. The following antibodies were used for immunophenotyping of PBMC: CD19-ECD (Beckman Coulter), PD-L1-PE-Cy7 (eBioscience), CD24-PE, CD38-FITC, HLA-DR-PE-Cy7, CD4-Pacific Blue, CD8-APC-H7, Lineage-1-FITC, CD11c- AF-700, HLA-ABC-PE-C7 and CD107a-PE-C5 (BD, Bioscience). HIV-specific CD8^+^ T cells were identified by binding to MHC-1-APC Dextramer® (Immudex) and HIV-infected CD4+ T cells were identified by binding to KC-57-PE antibody (Beckman Coulter). All samples were acquired on an LRSII (BD, Bioscience) flow cytometer and the data was analyzed using FlowJo software (Tree Star Inc).

### Functional Assays

Proliferation dye VPD450 (BD Bioscience)-labeled total or Breg-depleted PBMCs from HIV^+^ individuals were stimulated with HIV-peptide (NIH AIDS repository) pool spanning *nef*, *env*, *gag* and *pol* (2 ug/ml each) or 500 µM SAHA (Sigma). After 96 hours the frequencies of either CD8^+^CD107a^+^ (cytotoxic CD8^+^ T cells), infected CD4^+^ T cells (using KC57-Rd1, Beckman-Coulter, antibody that binds HIV-1 proteins 55, 39 33 and 24 KDa core) and proliferation of CD4+ T cells were determined by flow-cytometry. To determine the effect of PD-L1/PD-1 and IL-10/IL-10R interactions in Breg-mediated inhibition of CD4+ T cell proliferation, VPD450-labeled FACS-purified CD4^+^ T cells were activated using Anti-Biotin MACSiBead loaded CD2, CD3, and CD28 antibodies (Miltenyi, T Cell Activation/Expansion Kit) and IL-2 (20U, NIH AIDS repository), cultured with medium alone, with Bregs, with Bregs and IL-10R blocking antibody (20 ug/ml, BD Pharmingen), with Bregs and PD-1 blocking antibody (10 ug/ml, BD Pharmingen) or with Bregs with both IL-10R and PD-1 blocking antibodies. After 72 hours, proliferation was determined by flow cytometry.

### Staining of HIV-specific CD8^+^ T cells with HLA-A^*^0201-Restricted Peptide Complex

The frequency of antigen-specific CD8^+^ T cells was determined by binding to APC-labeled HLA-A2-restricted SL9 (SLYNTVATL) HIV-Gag epitope MHC-I-Dextramer (Immudex, Copenhagen, Denmark). Cells of HLA-A2 typed HIV^+^ individuals were washed twice with PBS, and incubated with 10 µl Dextramer for 10 minutes at room temperature, stained with antibodies and analyzed by flow-cytometry.

### LTR Real-Time PCR for HIV-1 DNA Quantification

RNA was isolated using Qiagen RNeasy Kit, according to the manufacturer's recommendations. Subsequently, cDNA was synthesized using the Quantitect Reverse Transcription kit (Qiagen, Valencia, CA). Real-time RT-PCR was performed using a Quantitect SYBR Green PCR kit (Qiagen) in a 7900HT Fast Real-Time PCR system (Applied Biosystems, Foster City, CA). Melting curve analysis was performed to ensure that the primers amplified the desired amplicon and that primer-dimers were absent. Primers used were: Long Terminal Repeats (LTR) mRNA forward 5′-TCAAGTAGTGTGTGCCCGTCTGTT-3′ and reverse 5′-AGCTCCTCTGGTTTCTCTTTCGCT-3′; and GAPDH mRNA forward 5′-CTTCAACGACCACTTTGT-3′ and reverse 5′-TGGTCCAGGGGTCTTACT-3′. Fold change in RNA expression was calculated by relative quantification using the comparative cycle threshold method. GAPDH expression was used as an endogenous control.

### Statistical Analysis

Results are expressed as mean ± standard error of the mean (SEM) or as indicated. GraphPad Prism software, version 5.03 was used for all statistical analysis. The statistical significance p value between group parameters was determined using either unpaired or paired tests (as indicated, with a confidence level of 95%). The statistical dependence between variables was calculated using the Spearman rank correlation analysis. p values of <0.05 were considered statistically significant.

## Supporting Information

Figure S1 **Detection of infected CD4+ T cells using the KC-57 antibody.** To determine the specificity of the KC-57 antibody, total or Breg-depleted PBMC from an HIV_ART_ subject, were supplemented with HIV-peptides (2 ug/ml of gag, pol, env and nef) and as a control PBMC were left unstimulated. After 4 days in culture, intracellular staining for KC-57 was performed and the frequency of stained infected cells determined by flow cytometry. Representative dot plots from 3 independent experiments are shown.
(TIF)

Figure S2 **Breg B-cells from HIV-infected subjects express high levels of Annexin V and low levels of Bcl-2.** To determine the cause of Breg loss in HIV-infected subjects by flow cytometry we assessed the frequency of determined Annexin V positive and intracellular Bcl-2 positive Bregs and non-Bregs (mature and memory B cells) in HIV-infected (**a,b**) and (**a**) HIV_NEG_ subjects. P values determined by Graphpad Prism software are indicated.
(TIF)
