# Supplementary figures and images for: Regulatory B Cells Inhibit Cytotoxic T Lymphocyte (CTL) Activity and Elimination of Infected CD4 T Cells after In Vitro Reactivation of HIV Latent Reservoirs
Source: PLoS One. 2014 Apr 16;9(4):e92934. doi: 10.1371/journal.pone.0092934 (PMC3989168; doi:10.1371/journal.pone.0092934)

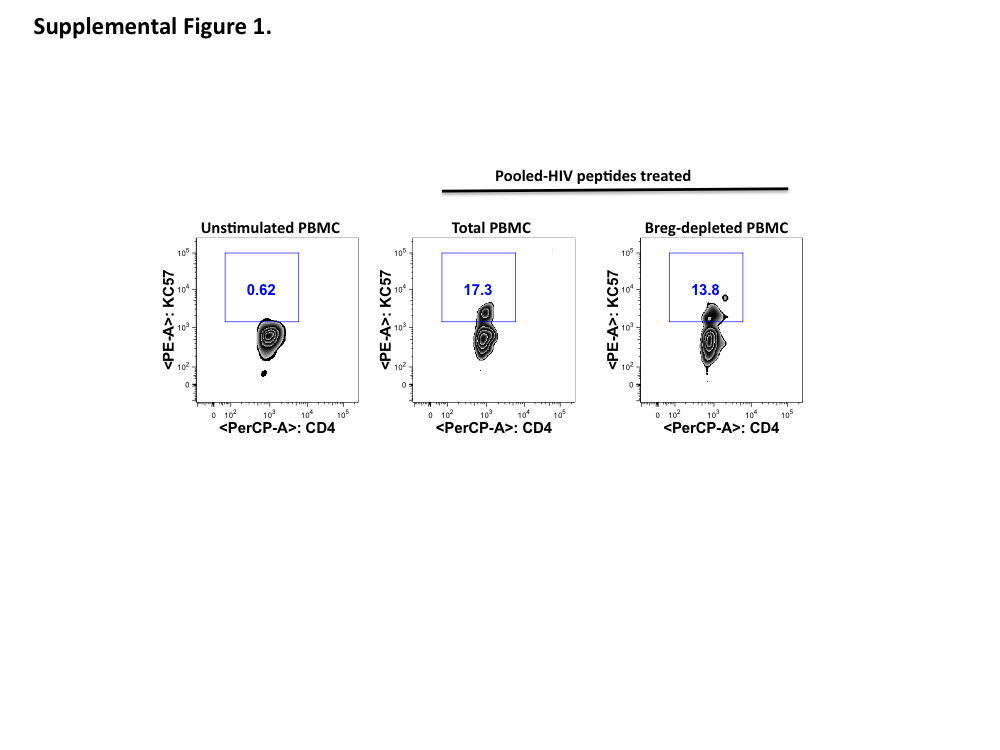

Supplement: Figure S2 — Breg B-cells from HIV-infected subjects express high levels of Annexin V and low levels of Bcl-2. To determine the cause of Breg loss in HIV-infected subjects by flow cytometry we assessed the frequency of determined Annexin V positive and intracellular Bcl-2 positive Bregs and non-Bregs (mature and memory B cells) in HIV-infected (a,b) and (a) HIVNEG subjects. P values determined by Graphpad Prism software are indicated. (TIF) [file pone.0092934.s002.tif]
